# On the use of certified reference materials for assuring the quality of results for the determination of mercury in environmental samples

**DOI:** 10.1007/s11356-016-7262-4

**Published:** 2016-08-15

**Authors:** Ewa Bulska, Agnieszka Krata, Mateusz Kałabun, Marcin Wojciechowski

**Affiliations:** 1 Faculty of Chemistry, Biological and Chemical Research Centre, University of Warsaw, Żwirki i Wigury 101, 02-089 Warsaw, Poland; 2 Inter-Faculty Interdisciplinary Doctoral Studies in Natural Sciences and Mathematics, University of Warsaw, Żwirki i Wigury 93, 02-089 Warsaw, Poland

**Keywords:** Quality of results, Environmental reference material, Mercury determination, Isotope dilution, Mass spectrometry

## Abstract

This work focused on the development and validation of methodologies for the accurate determination of mercury in environmental samples and its further application for the preparation and certification of new reference materials (RMs). Two certified RMs ERM-CC580 (inorganic matrix) and ERM-CE464 (organic matrix) were used for the evaluation of digestion conditions assuring the quantitative recovery of mercury. These conditions were then used for the digestion of new candidates for the environmental RMs: bottom sediment (M_2 BotSed), herring tissue (M_3 HerTis), cormorant tissue (M_4 CormTis), and codfish muscle (M_5 CodTis). Cold vapor atomic absorption spectrometry (CV AAS) and inductively coupled plasma mass spectrometry (ICP MS) were used for the measurement of mercury concentration in all RMs. In order to validate and assure the accuracy of results, isotope dilution mass spectrometry (IDMS) was applied as a primary method of measurement, assuring the traceability of obtained values to the SI units: the mole, the kilogram, and the second. Results obtained by IDMS using *n*(^200^Hg)/*n*(^202^Hg) ratio, with estimated combined uncertainty, were as follows: (916 ± 41)/[4.5 %] ng g^−1^ (M_2 BotSed), (236 ± 14)/[5.9 %] ng g^−1^ (M_3 HerTis), (2252 ± 54)/[2.4 %] ng g^−1^ (M_4 CormTis), and (303 ± 15)/[4.9 %] ng g^−1^ (M_CodTis), respectively. Different types of detection techniques and quantification (external calibration, standard addition, isotope dilution) were applied in order to improve the quality of the analytical results. The good agreement (within less than 2.5 %) between obtained results and those derived from the Inter-laboratory Comparison, executed by the Institute of Nuclear Chemistry and Technology (Warsaw, Poland) on the same sample matrices, further validated the analytical procedures developed in this study, as well as the concentration of mercury in all four new RMs. Although the developed protocol enabling the metrological certification of the reference value was exemplified by the determination of mercury in environmental samples, it could be considered as valid for any certification procedure required whenever new certified RMs are introduced.

## Introduction

The quality of results is an important issue in a whole range of scientific disciplines, in which chemical measurements are necessary. It has been recognized by those interested in environmentally related investigations that the quality of analytical results should be transparent and confident. Therefore, it is important to deliver accurate results and to be able to show their traceability (Bulska 2009).

Mercury was found to be one of the most toxic elements (Leermakers et al. 2005; Lubick 2009), causing several symptoms related to polyneuropathy mercurialis, neuroasthemia, tremor mercurialis, and other (Harada et al. 1999). Therefore, mercury pollution is an important issue that demands an ongoing development of analytical procedures to ensure its reliable determination. Certified reference materials (CRMs) are recognized to be an essential tool in assuring the accuracy and establishing the traceability of the results of measurements (Quevauviller et al. 1996). Thus, there is a need to introduce new CRMs in order to fulfill the demand of the environmentally oriented studies. So far, several analytical techniques were applied for the determination of mercury in various matrices and at various levels of concentration. The cold vapor atomic absorption spectrometry (CV AAS) is recognized to be one of the most widely used techniques for the determination of mercury. As the vapor of mercury is generated, it separates from the matrix, with a very high efficiency of analyte introduction. These features are crucial, when the concentration of mercury in the sample is low and the matrix is complex. Advantages of CV AAS resulted in its frequent use for the determination of mercury in biological samples (Shah et al. 2010; Torres et al. 2009), hair (Bruhn et al. 1994), slurries (Torres et al. 2009), water (Krata et al. 2002), and food packaging (Perring et al. 2001). Cold vapor generation was also combined with atomic fluorescence spectrometry (CV AFS) (da Silva et al. 2010; Li and Wang 2007) or inductively coupled plasma mass spectrometry (ICP MS) (Kenduzler et al. 2012; Knight et al. 1999).

The ICP MS has several advantages, such as high sensitivity and precision, excellent detection limits (below 0.01 μg L^−1^), selectivity, and possibility, to determine various isotopes of the element of interest. It was used for the determination of total mercury concentration in blood samples (McShane et al. 2008), marine organisms (Arslan et al. 2000; Falter and Ilgen 1997), food (Shuqin et al. 1999), sediments (Arslan et al. 2000; Falter and Ilgen 1997), water (Cattani et al. 2008; Soares dos Santos et al. 2009), and drugs (Wu et al. 2002). It should be mentioned, however, that the possible loss of mercury, due to its volatility during digestion or its ability for adsorption on the tubes’ walls of ICP MS sample introduction system, has to be considered. The last could be responsible for the memory effect; thus, several reagents (e.g., L-cysteine, thiourea, or gold chloride) were used to minimize it (Chen et al. 2000; Harrington et al. 2004; Li et al. 2006).

Isotope dilution mass spectrometry (IDMS) was used as a primary method of measurement, for the accurate determination of total mercury concentration (Heumann 2004; Quinn 1997; Taylor et al. 2001; Vogl and Pritzkow 2010). According to the definition of the Consultative Committee for Amount of Substance (Comite´ Consultatif pour la Quantite´ de Matie’re, CCQM), “a primary method is a method having the highest metrological qualities, whose operation can be completely described and understood, for which a complete uncertainty statement can be written down in term of SI units”. The IDMS approach is based on mathematical equation, which is well described. It consists of tabulated quantities, such as atomic weights and isotope abundances, as well as masses of sample and spike solution and, last but not least, isotopic ratios corrected for the mass discrimination effects (Vogl and Pritzkow 2010). Isotopic ratio in the mixture of sample and isotopically enriched standard is the only quantity, which is calculated from the ICP MS measurements of selected isotopes. Thereby, it is possible to calculate the concentration of an analyte with high accuracy and precision. Moreover, no quantitative recovery of analyte is required to obtain unbiased values after isotopic equilibrium is achieved. Although results obtained using IDMS approach provide traceability with SI unit system like the mole, the kilogram, and the second (Quinn 1997), it should be stressed that isotope dilution method is rarely used for routine measurements. The main field of its application is the certification of reference materials and enriched standards, where high accuracy, low uncertainty of measurement, and high quality of analytical results are the most important factors (Heumann 2004; Quinn 1997; Vogl and Pritzkow 2010).

Several approaches to environmental sample preparation before final measurements can be found in the literature; the microwave assisted digestion in closed vessels is commonly used for this purpose (da Silva et al. 2010; Falciani et al. 2000; Hoenig 2001). Nitric acid (V) alone is sufficient for digestion of organic reach matter; however, addition of hydrogen peroxide or hydrochloric acid can improve the performance and effectiveness of digestion (Aurelio and Arruda 2007; da Silva et al. 2010; Hoenig 2001; Knight et al. 1999). Soil and sediment matrices containing aluminosilicate require the addition of hydrofluoric acid or HCl (Arslan et al. 2000; Falciani et al. 2000). The use of aqua regia for the digestion of soils or sediments was also reported (Hoenig 2001; Smith 1993). In order to evaluate whether the selected digestion procedure does not cause the loss of some volatile elements, e.g., mercury, CRMs need to undergo the entire analytical procedure.

The aim of this work was to evaluate analytical procedures and measurement methods for the determination of mercury concentration in candidates for certified environmental reference materials obtained with metrological high quality. Two kinds of environmental materials (with inorganic and organic matrix) were used, and several digestion processes were assessed in order to receive quantitative recovery of mercury. Then, two analytical techniques such as ICP MS and CV AAS under different calibration strategies (external calibration, standard addition) were applied and compared. After that, IDMS measurements were performed. All the experiments were carried out towards certification of mercury concentration in candidates for CRMs.

The developed entire strategy for the metrological certification of the reference values could be however applied not only for the particular case described in this work. This protocol, which was proved to give metrological quality of the results, could be applied in any certification processes.

## Materials and methods

Trace analysis grade reagents nitric acid (Merck, Germany), hydrochloric and sulfuric acids (J.T.Baker, USA), hydrofluoric acid (Fluka, Germany), perchloric and tetrafluoroboric acids (Sigma-Aldrich, USA), as well as hydrogen peroxide (Fluka, Germany) were used for digestion of the samples. Sodium tetrahydridoborate (Sigma-Aldrich, USA) was used as reducing agent for CV AAS.

Two reference materials were used for the optimization of sample digestion: ERM-CC580 (Institute for Reference Materials and Measurements, IRMM, Belgium) estuarine sediment with the certified total mercury content of (132 ± 3) μg g^−1^ and ERM-CE464 tuna fish (IRMM, Belgium) with the certified total mercury content of (5.24 ± 0.10) μg g^−1^.

Isotopically enriched standard ERM-AE640 (IRMM, Belgium) enriched in 95.7 % of ^202^Hg with mercury concentration of (1.506 ± 0.011)·10^−5^ mol kg^−1^ was used for spiking the samples. ERM-AE639 Isotopic Reference Material (IRMM, Belgium) with mercury concentration (3.971 ± 0.015)·10^−5^ mol kg^−1^ was used for the correction of mass discrimination effects.

The 1641d mercury standard solution with concentration of (1.557 ± 0.020) mg kg^−1^ (NIST, USA) was used for calibration of ICP MS and CV AAS instruments, as well as for the standard addition ICP MS measurements. Standard solution of 1641d was diluted gravimetrically prior to use.

Rhodium standard solution and gold standard solution (Merck, Germany) each with concentration 1 g L^−1^ during measurements by calibration curve ICP MS were used.

The working standard solutions and blend solutions were prepared gravimetrically by appropriate dilution of the stock standard solutions using an analytical balance (Mettler Toledo, Switzerland).

Four candidates for CRMs with different matrix compositions identified as MODAS 2, MODAS 3, MODAS 4, and MODAS 5 (Institute of Nuclear Chemistry and Technology, IChTJ, Poland) (Table 1) were examined for the mercury concentration.

**Table 1:** List of candidates for certified reference materials

No.	ID	Matrix
1	MODAS 2: M_2 BotSed	Bottom sediment (from Vistula River near Włocławek city, Poland)
2	MODAS 3: M_3 HerTis	Herring tissue (*Clupea harengus*) (from the North Sea)
3	MODAS 4: M_4 CormTis	Cormorant tissue (*Phalacrocorax carbo)* (from Czech Republic)
4	MODAS 5: M_5 CodTis	Codfish tissue (*Gadus morhua*) (from Baltic Sea)

## Instrumentation

NexION 300D (PerkinElmer, USA) was used for the ICP MS measurements. The optimized working conditions used for mercury determination are listed in Table 2.

**Table 2:** ICP MS optimized working parameters

Parameter	Description
Spray chamber	Cyclonic, Scott type
Nebulizer	Quartz coaxial, type Meinhard
Plasma torch	Quartz
Sampler cone	Nickel
Skimmer cone	Nickel
Hyper skimmer cone	Aluminum
Plasma gas flow	16 L min^−1^
Nebulizing gas flow	1 L min^−1^
RF power	1600 W
Analog detector voltage	−1800 V
Pulse detector voltage	950 V
Lens voltage	−9 V
Dead time of detector	25 ns
Sample flow	1 mL min^−1^
Number of sweeps	5
Number of repeats	6
Integration time	150 ms
Monitored isotopes	^200^Hg, ^201^Hg, ^202^Hg

In case of IDMS approach, the signal intensities per replicate were corrected for dead time, instrumental background, and possible interferences prior to calculating an average isotopic ratio and its relative standard deviation. The dead time value and its associated standard uncertainty for every element were determined according to correction methods described by Nelms et al. (2001). The ratios *n*(^200^Hg)/*n*(^202^Hg) and *n*(^201^Hg)/*n*(^202^Hg) were selected for the mercury concentrations determination as all isotopes are free of significant spectral interferences. The mass discrimination effects were evaluated by measuring the ratio of *n*(^200^Hg)/*n*(^202^Hg) and *n*(^201^Hg)/*n*(^202^Hg) in the diluted ERM-AE639 standard solution containing around 5 ng g^−1^ of mercury and calculating the *K* ^200/202^ and *K* ^201/202^ factors from the ratio of the International Union of Pure and Applied Chemistry (IUPAC) data (Berglund and Wieser 2011) and the measured values. The mass discrimination correction was straightforward and resulted from the multiplication of the every measured blend ratio by the proper *K* ^200/202^ factor or and *K* ^201/202^ factor measured shortly after the blend. The analysis was performed according to the bracketing approach with an additional K-factor measurement at the end.

The procedural blanks, prepared in the same way as the samples or blend solutions, were determined by external calibration with three different concentrations of mercury, prepared by dilution of the respective standards.

FIMS 100 (PerkinElmer, Germany) atomic absorption spectrometer with a flow-injection unit was used for the determination of mercury. Samples were introduced by means of a 200-μL loop and reduction was performed by freshly prepared NaBH_4_ solution. Measurements were carried out in the peak-height mode. The optimized working conditions of cold vapor atomic absorption spectrometry (CV AAS) are presented in Table 3 (Krata et al. 2003).

**Table 3:** CV AAS optimized working parameters

Parameter	Description
Carrier gas	Argon
Carrier gas flow	75 mL min^−1^
Reduction agent	NaBH_4_, 0.025 % *w*/*v*
Rinse solution	HCl, 3 % *w*/*w*
Measurement mode	Peak area (integrated absorbance)

A microwave closed digestion unit (Ultrawave, Milestone, USA) was used for digestion of samples. Two programs were used for the optimization of digestion protocol of investigated samples (Table 4).

**Table 4:** Programs used for the digestion of examined samples

Parameter	Program I	Program II
Ramp time, min	15	25
Hold time, min	15	15
*T*_min_, °C	20	20
*T*_max_, °C	240	170
Pressure, bar	140	110

The main differences between digestion programs were as follows: in program I, ramp time was 15 min and the maximum temperature was 240 °C; in the case of program II, ramp time was extended to 25 min and maximum temperature was reduced to 170 °C.

## Results and discussion

### Determination of the moisture content

A correction for the moisture content was applied to all results. For the certified reference materials ERM-CC580 and ERM-CE464, the correction to dry mass was made by taking a separate portion of 100 mg and dried in an oven at 105 °C for 4 h, as recommended by the producer (IRMM). Samples of M_2 were dried at 105 °C for 24 h; all others samples were dried at 85 °C for 48 h, as recommended by the producer (IChTJ). Correction for dry mass was obtained from separate portions of the material mass of 0.2–0.4 g (ten subsamples from five bottles). The weighing and repeated drying were performed until constant mass was attained (0.0002 g difference between two successive weight measurements). The average moisture content was calculated and used for the mercury content correction of each sample. In case of ERM-CC580 and ERM-CE464, the moisture content was as follows: 5.1 and 7.5 %, respectively. For M_2, M_3, M_4, and M_5 the moisture content was found to be 4.3 %, 6.9 %, 5.1 %, and 5.6 %, respectively. According to producers’ instruction for the use of CRMs, the correction to dry mass should be made as the certified values are based on dry mass.

### Optimization of sample digestion conditions

The first part of this study was focused on the evaluation of the digestion procedures in order to obtain maximum recovery of mercury. CRMs of inorganic and organic matrix estuarine sediment (ERM-CC580) and tuna fish tissue (ERM-CE464) were used for this purpose. About 0.4 g of each material was weighted in the digestion vessel. After all reagents were added, the Teflon vessels were closed and put into the digestion unit. After careful optimization, two programs were used: program I and II, which were applied to the inorganic or organic matrix, respectively. Six different mixtures of acids were examined for the digestion of the ERM-CC580 (an example of inorganic matrix). The ICP MS calibration curve with Rh as internal standard was used for the determination of mercury in digested solutions. Composition of digestion mixtures and results calculated for various isotopes of mercury are presented in Table 5.

**Table 5:** Mercury concentration in ERM-CC580 certified reference material. Results expressed in microgram per gram (the extended uncertainty (*k* = 2) in μg g^−1^/%). Digestion conditions as in program I

No.	C_Hg_ (^200^Hg ± Uc)/Uc [%]	C_Hg_ (^201^Hg ± Uc)/Uc [%]	C_Hg_ (^202^Hg ± Uc)/Uc [%]	Composition of digestion mixture
1	(133 ± 2)/[1.5]	(133 ± 2)/[1.5]	(132 ± 2)/[1.5]	1 mL HF + 1 mL HCl + 3 mL HNO_3_
2	(130 ± 7)/[5.4]	(131 ± 7)/[5.3]	(129 ± 8)/[6.2]	3 mL HNO_3_ + 3 mL HF + 2 mL H_2_SO_4_
3	(118 ± 3)/[2.5]	(118 ± 3)/[2.5]	(117 ± 3)/[2.6]	3 mL HF + 2 mL H_2_SO_4_ + 1 mL H_2_O_2_
4	(139 ± 4)/[2.9]	(139 ± 4)/[2.9]	(137 ± 4)/[2.9]	3 mL HF + 2 mL H_2_SO_4_ + 3 mL HCl
5	(132 ± 9)/[6.8]	(133 ± 9)/[6.8]	(130 ± 10)/[7.7]	2 mL HNO_3_ + 2 mL HClO_4_ + 1 mL HF
6	(126 ± 15)/[11.9]	(126 ± 15)/[11.9]	(123 ± 15)/[12.2]	2 mL HNO_3_ + 1 mL HBF_4_ + 2 mL HClO_4_

Certified total mercury concentration (132 ± 3) μg g^−1^

Almost all obtained results (except when mixture of 3 mL HF + 2 mL H_2_SO_4_ + 1 mL H_2_O_2_ was used; —line 3 in the Table 5) were consistent with certified value for the total mercury concentration within its uncertainty in the ERM-CC580. Finally, the mixture of 1 mL HF + 1 mL HCl + 3 mL HNO_3_ with the highest accuracy and lowest uncertainty was selected for digestion of M_2 sediment sample.

For the digestion of the ERM-CE464 (an example of biological matrix), five different mixtures of acids were examined. The digestion program II was used. The ICP MS calibration curve with Rh as internal standard was used for the determination of mercury in digested solutions. Composition of digestion mixtures and obtained results are presented in Table 6.

**Table 6:** Mercury concentration in ERM-CE464 certified reference material. Results expressed in microgram per gram (the extended uncertainty (*k* = 2), μg g^−1^/%). Digestion conditions as in program II

No.	C_Hg_ (^200^Hg ± Uc)/Uc [%]	C_Hg_ (^201^Hg ± Uc)/Uc [%]	C_Hg_ (^202^Hg ± Uc)/Uc [%]	Composition of digestion mixture
1	(5.60 ± 0.09)/[1.6]	(5.60 ± 0.09)/[1.6]	(5.57 ± 0.08)/[1.4]	1 mL H_2_O_2_ + 4 mL HNO_3_
2	(4.90 ± 0.08)/[1.6]	(4.92 ± 0.07)/[1.4]	(4.86 ± 0.06)/[1.2]	1 mL H_2_O_2_ + 3 mL HNO_3_ + 3 mL HCl
3	(5.81 ± 0.08)/[1.4]	(5.83 ± 0.08)/[1.4]	(5.77 ± 0.06)/[1.0]	1 mL HNO_3_ + 3 mL HCl
4	(5.77 ± 0.14)/[2.4]	(5.80 ± 0.12)/[2.0]	(5.73 ± 0.12)/[2.1]	3 mL HNO_3_ + 1 mL HClO_4_
5	(5.23 ± 0.14)/[2.7]	(5.22 ± 0.12)/[2.3]	(5.18 ± 0.12)/[2.3)]	5 mL HNO_3_

Certified total mercury concentration (5.24 ± 0.10) μg g^−1^

It was found that addition of H_2_O_2_ or HCl or HClO_4_ to HNO_3_ did not improve the performance of digestion. Therefore, after evaluation of obtained results, finally, the concentrated solution of HNO_3_ was selected for the digestion of samples with organic matrix, M_3, M_4, and M_5.

### Optimization of digestion conditions for “MODAS” samples

The optimized digestion conditions for the inorganic and organic-like matrices were applied for the decomposition of “MODAS” samples (Table 4). The determination of mercury concentration was performed either with the use of the calibration curve (CV AAS or ICP MS) or with the standard addition (ICP MS). External calibration standards of mercury were at the level of 1, 5, and 10 μg L^−1^ and were prepared gravimetrically in 3 % HCl (for CV AAS) or in 2 % HNO_3_ (for ICP MS).

In the case of ICP MS measurements, rhodium was used as an internal standard. The intensities of three isotopes ^200^Hg, ^201^Hg, and ^202^Hg were monitored in “MODAS” samples without and with the addition of gold solution. By using the most abundant isotope ^202^Hg, the regression equation was *y* = 3090.9*x* with the correlation coefficient *R* ^2^ = 0.9998; precision was less than 2.5 % for the investigation concentrations range; the limit of quantification and detection were 0.48 μg kg^−1^ and 0.18 μg kg^−1^, respectively. In order to evaluate any possible matrix effects, the standard addition was also applied. To each sample, a set of three known amounts of mercury standards was added gravimetrically and proportionally to the mercury concentration in sample. The precision was less than 2 % for the investigated concentrations range. The results of mercury content in all samples were identical (within their uncertainty) for all monitored isotopes. Thus, in Table 7, only the results for the most abundant isotope ^202^Hg were listed.

**Table 7:** Mercury concentration in “MODAS” materials with the use of ICP MS and CV AAS. Results expressed in nanogram per gram (the extended uncertainty (*k* = 2), ng g^−1^/%)

Method	ICP MS calibration curve (no addition of Au)	ICP MS calibration curve (with addition of Au)	ICP MS standard addition	CV AAS calibration curve peak area mode
Material	C_Hg_ (^202^Hg ± Uc)/Uc [%]	C_Hg_ (^202^Hg ± Uc)/Uc [%]	(C_Hg_ ± Uc)/Uc [%]	(C_Hg_ ± Uc)/Uc [%]
M_2 BotSed	(399 ± 15)/[3.8]	(863 ± 47)/[4.3]	(883 ± 25)/[2.9]	(951 ± 67)/[7.1]
M_3 HerTis	(116 ± 6)/[5.5]	(239 ± 16)/[5.6]	(247 ± 8)/[3.5]	(245 ± 21)/[8.7]
M_4 CormTis	(2108 ± 89)/[4.2]	(2080 ± 82)/[4.0]	(2284 ± 130)/[5.7]	(2238 ± 81)/[3.6]
M_5 CodTis	(241 ± 17)/[6.9]	(294 ± 21)/[8.6]	(314 ± 16)/[5.0]	(297 ± 14)/[4.6]

The measurements performed by ICP MS were conducted first with the use of external calibration, when rhodium was added as an internal standard. Next, the standard addition ICP MS was applied. The results obtained with ICP MS by the standard addition method were significantly higher, compared to those obtained with the use of the external calibration (except M_4, results were in agreement within uncertainty). Therefore, it was decided to add gold solution as preservative agent during the digestion step. This work focused on the development and validation of methodologies for the accurate determination of mercury in environmental samples and its further application for the preparation and certification of new reference materials. Two certified reference materials ERM-CC580 (inorganic matrix) and ERM-CE464 (organic matrix) were used for the evaluation of digestion conditions assuring the quantitative recovery of mercury. These conditions were then used for the digestion of new candidates for the environmental reference materials (RMs): bottom sediment (M_2 BotSed), herring tissue (M_3 HerTis), cormorant tissue (M_4 CormTis), and codfish muscle (M_5 CodTis). Cold vapor atomic absorption spectrometry (CV AAS) and inductively coupled plasma mass spectrometry (ICP MS) were used for the measurement of mercury concentration in all RMs. In order to validate and assure the accuracy of results, isotope dilution mass spectrometry (IDMS) was applied as a primary method of measurement, assuring the traceability of obtained values to the SI units: the mole, the kilogram, and the second. Results obtained by IDMS using *n*(^200^Hg)/*n*(^202^Hg) ratio, with estimated combined uncertainty, were as follows: (916 ± 41)/[4.5 %] ng g^−1^ (M_2 BotSed), (236 ± 14)/[5.9 %] ng g^−1^ (M_3 HerTis), (2252 ± 54)/[2.4 %] ng g^−1^ (M_4 CormTis), and (303 ± 15)/[4.9 %] ng g^−1^ (M_CodTis), respectively. Different types of detection techniques and quantification (external calibration, standard addition, isotope dilution) were applied in order to improve the quality of the analytical results. The good agreement (within less than 2.5 %) between obtained results and those derived from the Inter-laboratory Comparison, executed by the Institute of Nuclear Chemistry and Technology (Warsaw, Poland) on the same sample matrices, was achieved. It has been known that gold will amalgamate mercury, thus preventing other side reactions as well as overcoming the problem of memory effect (Allibone et al. 1999). For this purpose, the 100-fold excess of gold standard solution (proportional to the mercury content in “MODAS” samples) was gravimetrically added to each sample and procedural blank, before the digestion step, and then digestion procedure was applied. The gold standard solution was also added to the mercury standard solutions: 1, 5, and 10 μg L^−1^ prepared gravimetrically, as to reach 100, 500, and 1000 μg L^−1^ of gold in the standard solution, respectively. With this approach, the results of mercury concentrations achieved by the calibration curve ICP MS were in agreement within uncertainty with results obtained by standard addition method.

CV AAS was used in order to assure the accuracy of the results of mercury concentrations obtained by ICP MS. The regression equation was *y* = 0.0094*x* with the correlation coefficient *R* ^2^ = 0.9996; precision was less than 3 % for the investigation concentrations range; limits of quantification and detection of 0.54 μg kg^−1^ and 0.25 μg kg^−1^ were received, respectively. The measurement conditions of CV AAS, as listed in Table 3, were validated by measuring the concentration of mercury in the ERM-CE464. Calculated concentration of mercury in the ERM-CE464 was in good agreement with the certified value. The results obtained for “MODAS” samples (Table 7) were found to be in a good agreement within their uncertainty with the results, when the calibration curve with gold standard solution was added and the standard addition ICP MS were applied.

### Isotope dilution mass spectrometry as a primary method of measurement for mercury determination

In order to evaluate the accuracy of the results presented in Table 7, IDMS was applied as a primary method of measurement. The optimal amount of isotopically enriched standard was calculated to alter natural isotopic composition of mercury (from 0.77 till around 0.2) in the samples. The ERM-AE640 isotopically enriched standard (95.7 % abundance for ^202^Hg) was used for this purpose (Certificate of ERM-AE640 2016). Established amounts of isotopically enriched standard (0.45 g, 0.16 g, 1.7 g, and 0.26 g) were added gravimetrically to the “MODAS” samples (to M_2, M_3, M_4, and M_5, respectively). Two isotopic ratios were calculated: *n*(^200^Hg)/*n*(^202^Hg) and *n*(^201^Hg)/*n*(^202^Hg) by monitoring most abundant isotopes of mercury by ICP MS. Special attention was paid to assure that the isotopic equilibrium was achieved, after mixing of the sample and spike during digestion. In the case of M_2, results were corrected mathematically due to tungsten presence in the sample, which formed ^184^W^16^O^+^ and ^186^W^16^O^+^ ions. In the other three materials, the content of tungsten was sufficiently low; thus, interferences were not observed. It can be seen in Table 8 that mercury concentrations calculated from two different isotopic ratios with the use of basic IMDS equation (Heumann 2004; Vogl and Pritzkow 2010) were in very good agreement within uncertainty values. It should be stressed that mercury concentrations in “MODAS” reference materials reported by the organizer (Institute of Nuclear Chemistry and Technology (Warsaw, Poland)) of Inter-laboratory Comparison, ILC (Table 8), are consistent with mercury concentrations obtained in this study with the use of different analytical techniques and calibration strategies: the calibration curve and the standard addition ICP MS, CV AAS technique, and a direct IDMS.

**Table 8:** Mercury concentration in MODAS reference materials: (i) calculated from *n*(^200^Hg)/*n*(^202^Hg) and *n*(^201^Hg)/*n*(^202^Hg) ratios; (ii) from Inter-laboratory Comparison (ILC). Results are expressed in nanogram per gram (the extended uncertainty (*k* = 2), ng g^−1^/%)

Material	IDMS		C_Hg_ ± Uc (*k* = 2) ILC [%]
	*n*(^200^Hg)/*n*(^202^Hg) [%]	*n*(^201^Hg/*n*(^202^Hg) [%]	
MODAS 2	(916 ± 41)/[4.5]	(899 ± 40)/[4.4]	(884 ± 53)/[6.0]
MODAS 3	(236 ± 14)/[5.9]	(237 ± 14)/[5.9]	(227 ± 21)/[9.2]
MODAS 4	(2232 ± 54)/[2.4]	(2234 ± 54)/[2.4]	(2200 ± 140)/[6.4]
MODAS 5	(303 ± 15)/[4.9]	(301 ± 15)/[4.9]	(310 ± 22)/[7.1]

The extended uncertainty of measurement results was estimated for entire IDMS procedure. All factors influencing the final results, as well as isotopic equilibrium, were systematically investigated. This included the procedural blank, the moisture content in “MODAS” samples, and all factors affecting the blend ratio measurements (instrumental background, spectral interferences, dead time, and mass discrimination effects as well as the repeatability of measured isotopic ratios). For the evaluation of uncertainties of measurement values, the GUM Workbench 2.4 (Metrodata GmbH 2016) software was used. Budget of uncertainty for IDMS measurements is presented in Table 9. It can be seen that concentration of mercury in blank was a dominant source of uncertainty in all studied “MODAS” materials, what means that applied measurement conditions confirmed the metrological quality of obtained results.

**Table 9:** Main sources of uncertainty of mercury concentration in “MODAS” materials by means of IDMS

Element of budget	MODAS 2 (%)	MODAS 3 (%)	MODAS 4 (%)	MODAS 5 (%)
	88.0	91.8	92.3	78.7
Dead time	2.3	1.7	2.5	5.8
Concentration of ERM AE640 standard	2.5	1.7	1.6	4.4
% abundance of ^198^Hg in the standard	1.8	0.5	0.4	1.3
% abundance of ^199^Hg in the standard	0.4	0.6	0.5	1.5
% abundance of ^200^Hg in the standard	1.2	0.4	0.4	1.1
% abundance of ^202^Hg in the standard	1.2	0.4	0.4	1.0
% abundance of ^204^Hg in the standard	0.6	0.3	0.3	0.7
Determination of isotopic ratio in mixture 1	0.2	0.4	0.4	0.8
Determination of isotopic ratio in mixture 2	0.6	0.8	0.0	1.6
Determination of isotopic ratio in mixture 3	0.4	0.4	0.2	0.4
Water content	0.8	0.6	0.1	0.3

## Conclusions

In this work, analytical methodologies for the accurate determination of mercury in environmental samples of inorganic or organic origins were developed. Subsequently, developed conditions were applied for the preparation of new reference materials with the proposed certified values for mercury. In order to assure the accuracy of the results, two certified reference materials (ERM-CC580 and ERM-CC464) were used. Recoveries close to 100 % of the certified values, within uncertainties, were achieved with the use of optimized digestion procedures for inorganic and organic matrix, respectively. The same digestion conditions were used for new reference materials called “MODAS” CRMs. The determination of mercury was then performed with the use of CV AAS, ICP MS (with external calibration or standard addition strategy), and finally validated by IDMS as a primary method of measurement. The mercury concentrations obtained by means of ICP MS (by external calibration in the presence of gold solution) and CV AAS were in good agreement within their uncertainties. On the base of those results, the optimal amount of isotopically enriched standard was calculated for alteration of natural mercury isotopic ratios in “MODAS” samples and IDMS for mercury determination was applied. Application of IDMS enabled traceability of obtained results to SI units and assured the accuracy of measurements. Water content in all materials used in this study was established by dry mass correction. The determined concentrations of mercury in “MODAS” reference materials were cross-validated with records from Inter-laboratory Comparison. The difference between obtained results in this study and those derived from ILC on the same sample matrices was less than 2.5 % and further validated the developed methodologies for the accurate determination of mercury in environmental samples. It should be highlighted that the obtained results by IDMS for mercury concentrations were used later as a contribution of the Biological and Chemical Research Centre in the frame of certification process of “MODAS” reference materials organized by the Institute of Nuclear Chemistry and Technology.

The methodology developed within this study was used for certification of mercury concentration in selected candidates for environmental reference materials. Despite the measurement technique, the limit of detection was found to be around 0.2 μg kg^−1^. The lowest extended uncertainty of results, below 5.9 %, was obtained when the standard addition ICP MS or IDMS was used which was lower than the values provided by the organizer of respective ILC. Thus, the developed protocols could be recommended as a generally valid for the certification of reference materials of environmental origin.
